# Effect of Compaction Pressure and Moisture Content on Post-Agglomeration Elastic Springback of Pellets

**DOI:** 10.3390/ma14040879

**Published:** 2021-02-12

**Authors:** Jakub Styks, Adrian Knapczyk, Bogusława Łapczyńska-Kordon

**Affiliations:** Department of Mechanical Engineering and Agrophysics, University of Agriculture in Krakow, Balicka 120, 31-120 Kraków, Poland; adrian.knapczyk@urk.edu.pl (A.K.); boguslawa.lapczynska-kordon@urk.edu.pl (B.Ł.-K.)

**Keywords:** pressure compaction, moisture content, cup plant, Virginia mallow, giant miscanthus, perennial biomass, compaction, pressure, springback, biomass, pellets

## Abstract

Renewable energy sources (RES) represent an increasing share of global energy production. Biomass has the highest potential of all RES. Biomass is used to produce solid biofuels, liquid biofuels, and gaseous biofuels. One of the main directions of research on solid biofuels is to optimize the agglomeration process. The main factors determining the characteristics of the final product in the production of pellets are process and material parameters. Process parameters include compaction pressure, temperature, and geometry of the matrix channel. The parameters of the material are the type of biomass, moisture content, degree of fragmentation, and method of preparation of the material (e.g., drying). The process of pressure compaction is always associated with the negative phenomenon of elastic springback. The aim of this work was to check the influence of compaction pressure and material moisture content on the springback value. The research was conducted on three materials (giant miscanthus, cup plant and Virginia mallow), using four different pressures (131, 196, 262, and 327 MPa) and three different moisture levels (8, 11, and 14%). For all material springback values, the range was 9–16%. Statistical analysis showed that for all plants tested, the effects of compaction pressure and moisture content significantly affected the elastic springback value. Areas of high value springback in the pattern of process parameters were determined.

## 1. Introduction

In recent years, we have seen an increase in energy consumption directly related to population growth. The main energy source is fossil fuels. Nevertheless, renewable energy sources (RES) constitute a growing share of world energy production and, consequently, the interest of researchers in this subject is growing. The increasing demand for energy and environmental protection has led to an increased interest in research into RES—sun, wind, geothermal energy, water, sea, and biomass [1,2].

Many studies have shown that biomass has the highest energy potential of all RES sources. Biomass can be a raw material for the production of solid, liquid, or gaseous biofuels. Solid biofuels can be defined as untreated and processed biomass. Biomass sources can be energy crops, waste from agricultural production, wheat seeds, waste wood, peat, or waste from agri-food processing [3,4,5].

The main research directions in the production of solid biofuels are searching for new production materials (e.g., exotic plants), determination of physical-mechanical, chemical and energy properties of selected materials, analysis and optimization of the supply chain, technologies of biomass processing to biofuels (e.g., torrefaction and agglomeration), and optimization of the biofuel production process [6,7,8,9,10].

Methods for processing biofuels can be divided into biological conversion, physical conversion, chemical conversion, and thermo-chemical conversion. These methods are used to improve the physico-mechanical and chemical parameters of biofuels. The resulting products have various applications, such as biofuels, fertilization, soil remediation, and biosorbents [11]. As a result of processing, the products can be used directly or serve as a raw material for further processing [12,13,14].

One of the main technologies of biomass processing is physical conversion, or more precisely, agglomeration. The agglomeration process can be divided into pressurized and unpressurized. In the production of solid biofuels only pressure agglomeration is used. Depending on the final product (briquette or pellets), different production technologies are used. In the case of production of briquettes two main methods of agglomeration can be distinguished, namely continuous (briquetting screw presses) and cyclic (briquetting presses with open and closed chamber). In the case of pellets, granulators with a ring or flat matrix are used. As a result of pressure agglomeration, we obtain a fuel unit unified in terms of shape, moisture, density, and calorific value in the form of a briquette or pellet. Pellets are the most processed fuel and have the highest operational potential (dimensional uniformity facilitating the automation of the process of inflow and combustion) [15,16,17,18,19].

The main factors determining the characteristics of the final product when producing pellets and briquettes are process and material parameters. Process parameters include agglomeration pressure, temperature, and geometry of the matrix channel or compression chamber. Material parameters are type of biomass, moisture content, degree of fragmentation, and method of raw material preparation (e.g., drying the material) [20,21,22]. The main quality parameters depending on process and material factors are mechanical durability (DU) and specific density. The process of pressure compaction of biomass is intended to obtain the specific density (DE) and bulk density (BD) as well as the mechanical durability at appropriate levels, and the minimum thresholds are defined by the appropriate standards. Unified bulk density (or specific density) guarantees similar values of energy density of fuel—regardless of the raw material used. On the other hand, high mechanical durability guarantees safe and non-abrasive use of the fuel. The lack of fine fraction and dust in that kind of fuel means the lack of dust during logistic operations, both at the stage of delivery and storage, but mainly during the loading of tanks and containers of combustion systems [23,24,25,26].

These parameters are not related to the energy properties of the product which depend directly on the type of raw material. However, they influence transport, storage, or combustion process. Achieving the threshold values of quality parameters required by the standards ensures the correct flow of these processes.

An undesirable phenomenon causing a decrease mechanical durability and specific density of compacted fuel (both pellet and briquette) is their expansion or “springback effect” [27,28,29]. It is formed after the compaction pressure has ceased and generally causes the particles of the material forming the agglomerate to move away, which reduces the number of contact points, reduces the contact area and, as a result, weakens the intermolecular connections formed during the compaction. This phenomenon always accompanies the process of biomass pressure compaction and is caused by the internal structure of the biomass and also the structure of the particle deposit.

The lignocellulosic biomass cell walls are made of natural composite, the skeleton of which is formed by cellulose fibers connected by a network of hemicellulose and the whole is joined by lignin. In a spatial aspect the ligneous cells are mostly in the form of a capillary, porous honeycomb structure. This shape, in combination with the lignocellulose composite, gives mechanical durability to the cellular assemblies that form the plant’s skeleton (tree trunks and branches, grass stalks, and herbaceous plant shoots). This gives strength to the plant, but during the agglomeration it induces stress in the raw material [30].

In the processes of pressure agglomeration, the specific density of the product is aimed at similar values regardless of the raw material. By compacting the shredded material, we reduce both the cavities between the raw material particles and the cavities inside the tissue. In order to maintain the degree of porosity reduction after the compaction pressure has stopped, mechanisms bonding the particles together are activated [31,32].

Major mechanisms keeping particles connected are van der Waals forces, fiber interlocking, covalent and hydrogen bonds, and lignin–glass transition [33,34,35,36,37]. The lignin bridges are stronger than the covalent and hydrogen bonds. The temperature of lignin activation is more than 110 °C, but it can be lowered by the right moisture content (MC) in material, so temperature without moisture will cause the bonding effect, but only at high temperatures [29]. Therefore, for biomass compaction processes, the presence of water in the material is always required. Moisture content in range of 8–20% was proposed by Kalian and Morey [38] however, the range used in practice is 8–15% [39,40,41]. The technical moisture content of the raw material is also limited by quality standards. For the highest quality class, they require woody pellet moisture below 10% [42] and non-woody pellets and briquettes moisture below 12% [43,44,45]. Even taking into account a 2–3% drop in moisture during the production process, the upper raw material moisture value is at 14–15%.

Therefore, during the compaction of lignocellulosic biomass, the goal should be to overcome its elasticity and plasticization, especially of lignin. This will cause stable deformation of the densified material particles and the occurrence of strong bonds between them.

Some factors reduce the elasticity of the material and cause an increase in its plasticity, which diminishes the phenomenon of springback (SP) but does not remove it completely. Such a factor is in addition to the previously-mentioned moisture content and temperature that plasticizes the biomass particles and activates binders e.g., lignin. The influence of these factors is closely related to the raw material and must be individually selected for [28]. Other factors that can affect springback are the degree of fragmentation, the particle shape generated by different grinding systems, or the drying temperature, which affects grindability, which is probably related to material brittleness [46].

The investigation of the springback phenomenon is a relatively new topic [47] although this effect has been observed in other studies of biomass agglomeration [27]. As Frodeson indicates, biomass containing more xylans is characterized by lower springback. Wróbel [48] determined the impact of pressure on the elastic springback value for a wide range of dry (MC about 0%) woody and herbaceous biomass (15 species). The value of obtained elastic springback ranged from 35% for pine to 69% for birch, however most of the material had an elastic springback in the range of 40–50%. Dhamodaran and Afzal [29] determined the effect of moisture and temperature on SP of soft and hard wood. Among the examined factors (MC of 8 and 10%, temperature of 60 and 70 °C, and pressure of 139.3 and 159.2 MPa), the strongest impact on the increase of springback was caused by the increase of moisture content. Tested temperature levels did not cause change in springback value. Increase in pressure value, in several measurement variants, caused a slight increase in springback. It can be assumed that temperatures up to 70 °C do not influence the elastic springback. Temperature causes plasticization of lignin when it is above 110 °C [49,50]. This plasticization can occur at lower temperatures of 75–90 °C on condition that water is contained in the material [51] and that there is high pressure level [50]. Wheat straw lignin–glass transition occurs at 53–63 °C and MC of 8% [34]. Low levels of moisture in torrefied biomass increase the glass transition temperature [33]. In corn stover and switchgrass the mean glass transition temperature is 75 °C at 10–20% moisture content [52].

Accordingly, moisture at the right level is necessary for proper biomass compaction process and high temperature is a supporting factor, but not always applicable (e.g., some briquetting methods).

Another factor making it difficult to compare results from different tests (even if the materials are the same) is the particle size distribution (PDS) of raw material. It is often varied or not stated, although, as the research results shows, it has an impact on the springback [53].

Thus, many material and process factors affect the elastic springback, but pressure (P) and moisture (MC) are the main, primary factors which are almost always present in biomass agglomeration. Recognizing and understanding the process of springback should start with recognizing the influence of the material (treated in general with possible composition determination), moisture content, and pressure. The influence of other factors and the interactions between them is the next step.

Therefore, the aim of the study was to determine the influence of compaction pressure and moisture content on the expansion of test pellets produced from selected types of biomass. The influence of PSD, raw material dry temperature or grinding systems, and compaction temperature was intentionally omitted.

## 2. Materials and Methods

Plants used as the test material were obtained from the energy plant plantation located in plants located in University of Agriculture in Krakow. The chosen material was shoots of giant miscanthus, *Miscanthus × giganteus* Greef et Deu, cup plant *Silphium perfoliatum* L., and Virginia mallow *Sida hermaphrodita* (L.) Rusby. Shoots were harvested after the growing season, in November 2018.

**Figure 1:**
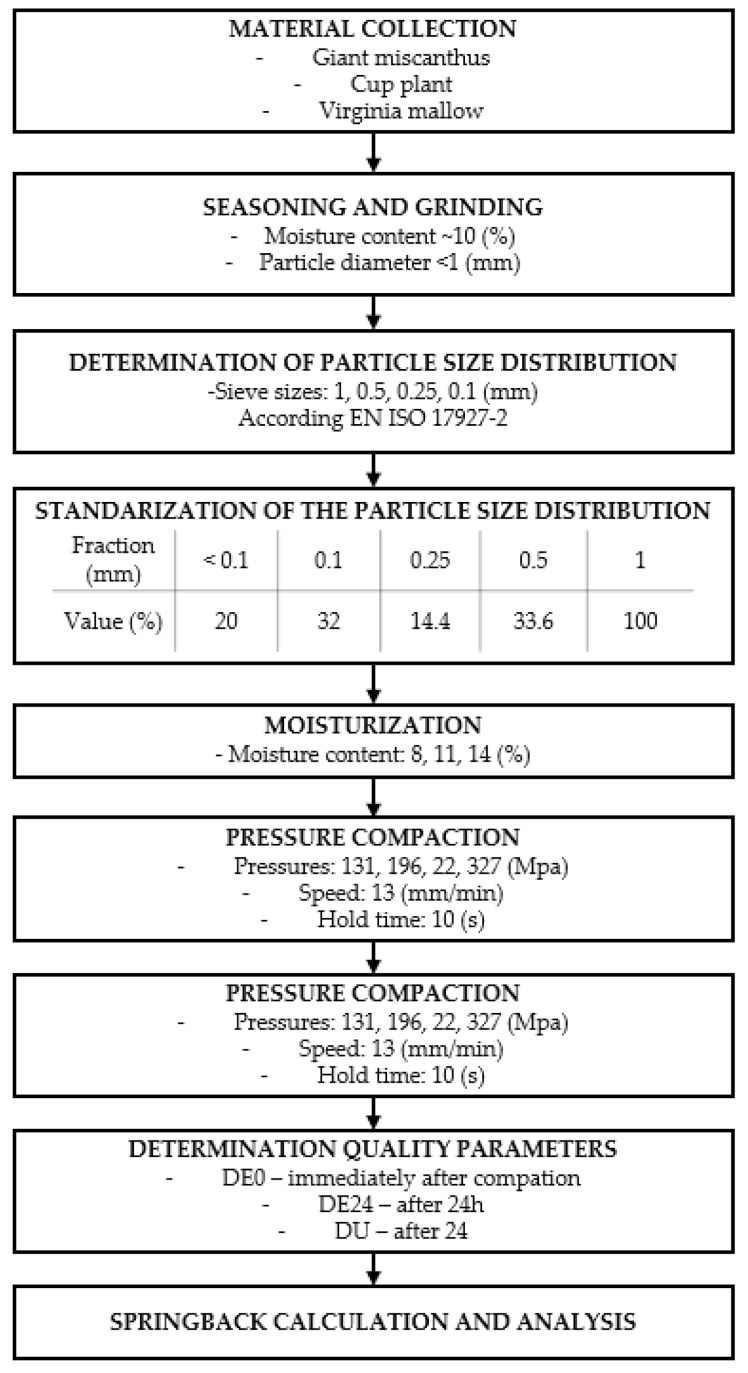
Diagram of research methodology.

The research was carried out according to the flowchart shown in Figure 1. After harvesting, the material was seasoned and then grounded to a grain size of d < 1 mm. In order to omit the influence of PSD on the obtained results, material of standardized grain composition proposed by Wróbel [48] was prepared (Figure 1).

Sample moisture content was maintained at three different levels—8, 11, and 14%. The lower value corresponds to the range proposed by Kalian and Morey (8–20%) [38]. The proposed range is more often 8–15% [24]. Therefore, the upper range was assumed to be 14%, but in practice pellets with this moisture content do not meet the quality standards.

The process of pressure was conducted at 131, 196, 262, and 327 MPa (which corresponds to force equal to 14.82, 22.17, 29.63, and 36.98 kN). The pressure range widely covers the pressure used in briquetting and pelletizing. The tests were carried out on single pellet station diameter 12 mm. Hold time was established at 10 s.

Each sample was made in three repetitions, which finally gave 108 samples. Determination of specific density (DE) was carried out immediately after leaving the compaction chamber and 24 h after the production of pellets. Then, based on the acquired data, the post-agglomeric elastic springback (SP) was determined. Details of the research process are presented in the Figure 1.

A detailed description of research methods applied to the stage “pressure compaction” (Figure 1) are described in in a previous work [24].

After the production of the samples, their geometry was determined. The measurements were repeated after 24 h, which made it possible to calculate the post-agglomeration springback SP [54]. The value of this parameter determines the extent to which the characteristics of the raw material, as well as the factors and conditions of the densification process, have an influence on the produced granules. The expansion is a percentage increase in the volume of the *V_k_*_24_ granule in relation to the initial volume *V_k_*.

$$\mathrm{SP}=\frac{(V_{k24}-V_{k0)}}{V_{k0}}\times 100$$

where SP is elastic springback (%), *V_k_*_0_ is initial granule volume (mm^3^), and *V_k_*_24_ is the volume of granules after 24 h of production (mm^3^).

The collected results were analyzed statistically. A detailed statistical analysis was made for two hypotheses:

− Differences in the moisture content of the material have a significant impact on elastic springback.
− Differences in the compaction pressure significantly affect elastic springback.

In this paper two-way analysis of variance (ANOVA) was carried out. Normality of decomposition was checked (Shapiro–Wilk test). For all cases, the distribution was normal. Then the assumption of the equality of variance was also checked (Brown–Forsythe test). For all cases, this equality has been met. The next step was to carry out one-way ANOVA and post-hoc analysis (Scheffé’s test), which indicated between which groups there were statistically significant differences.

In the next step was correlation analysis and was also performed for two variants—specific density vs. elastic springback and mechanical durability vs. elastic springback.

## 3. Results and Discussion

Studies have shown a relationship between moisture content and increasing compaction pressure and elastic springback of produced pellets.

### 3.1. Statistical Analysis

**Figure 2:**
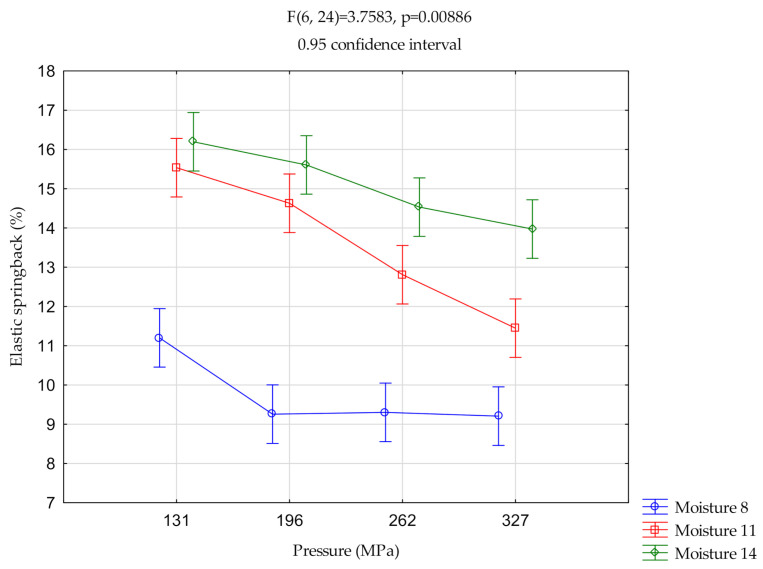
Two-way analysis of variance (ANOVA)—miscanthus.

**Figure 3:**
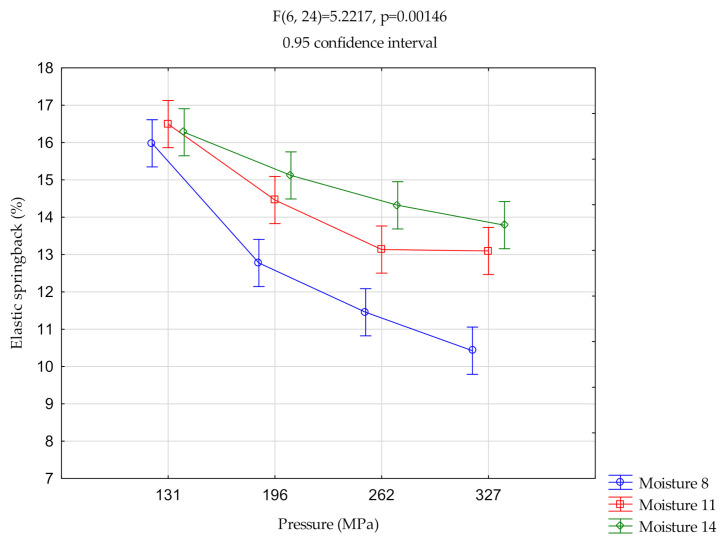
Two-way analysis of variance (ANOVA)—silphium.

**Figure 4:**
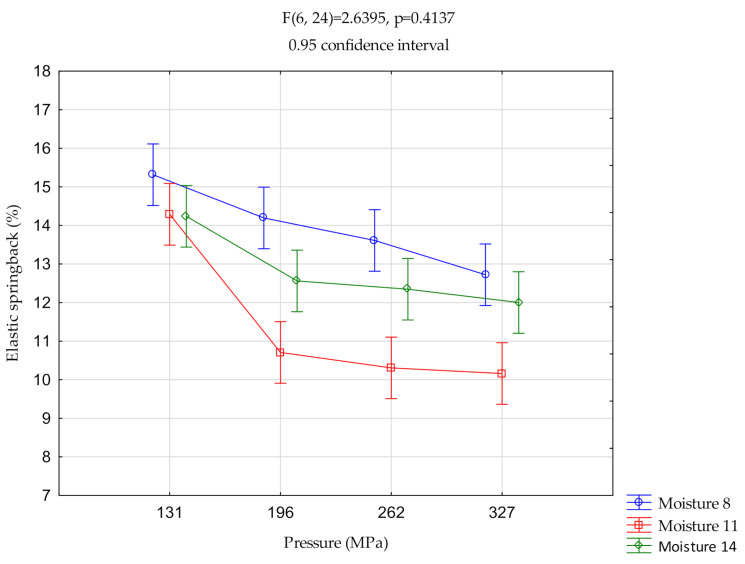
Two-way analysis of variance (ANOVA)—sida.

Table 1, Table 2 and Table 3 show three-way ANOVA results for the significance level α = 0.05. The individual values of mean were SS, sum of squares; df, degrees of freedom; MS, mean square; F Value, F test value; *p* value, probability value. The *p* value indicator shows the probability of obtaining results as extreme as the observed results of a statistical hypothesis test. The F value indicator is the value of a statistical hypothesis test value. The results are presented for single factors (pressure and moisture) and for combined factors (e.g., pressure × moisture).

(Figure 2, Figure 3 and Figure 4) show the relationships of elastic springback vs. pressure and vs. moisture. The centers of the ranges represent the mean value. Vertical bars represent 0.95 confidence intervals.

#### 3.1.1. Miscanthus

For pressures P131, P196, and P262, the elastic springback value at 8% moisture content was significantly different from the remaining moisture content. At P327, there were significant differences between all the moisture contents. At 8% and 11% moisture, the springback elastic value for P131 differed significantly from that for other pressures.

In this case, an increase in moisture content from 8 to 11 I 14 resulted in a significant increase in elastic springback. For a pressure of 131 MPa, R increased from about 11% to 15.5–16.5% and at 196 MPa, from 9.5% to 14.5–15.5%. Further increase of the pressure caused smaller differences between the values of the obtained elastic springback.

#### 3.1.2. Silphium

For pressures P196, P262, and P327, the elastic springback value for 8% moisture content differed significantly from that for the remaining moisture content. The springback elastic value at P131 differed significantly from the other values at all tested moisture contents.

In this case, as the moisture content increased, the elastic springback value increased. The increase in pressure caused an increase in the differences between the elastic springback values for the individual moisture contents. At 131 MPa there were no significant differences between the elastic springback values for all the moistures. At 327 MPa the elastic springback increased from 10.5% to about 14%.

#### 3.1.3. Sida

The elastic springback value for pressures P196, P262, and P327 was significantly different for 11% moisture. For moisture contents of 8%, 11%, and 14%, the springback elastic value was significantly different at P131.

In this case, we observed a significantly different relationship compared to the previous materials. At a moisture content of 8% the highest elastic springback occurred for all pressure values. At a moisture content of 11, a minimum elastic springback value was obtained after a pressure of 196 MPa. For a pressure value of 131 MPa for all moisture values, there are minimum differences between the elastic springback values. At 196 MPa, the elastic springback value increased from 10.5% to about 14%.

#### 3.1.4. Elastic Springback vs. Pressure and Moisture Content

**Figure 5:**
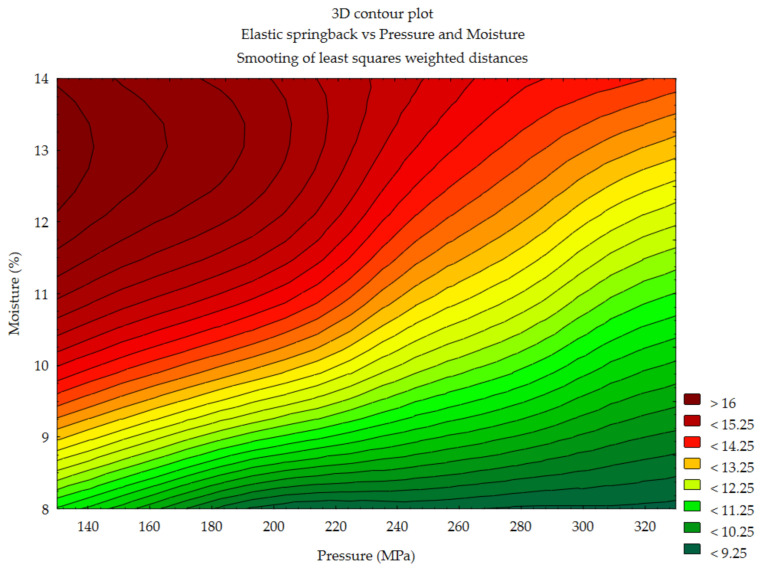
Elastic springback vs. pressure and moisture—miscanthus.

**Figure 6:**
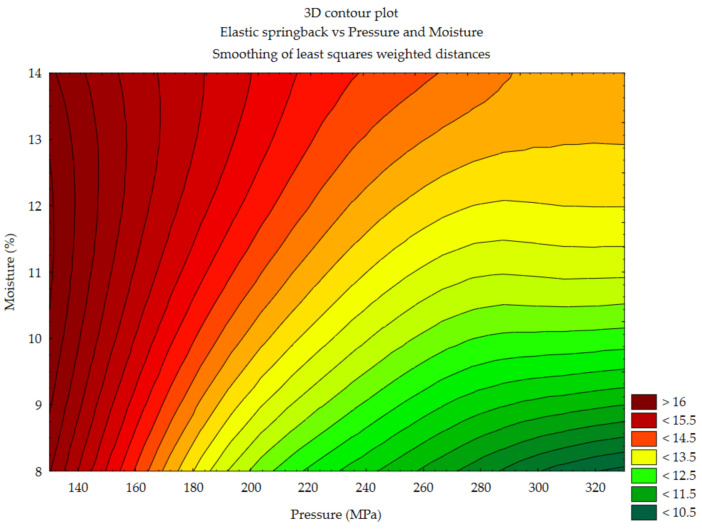
Elastic springback vs. pressure and moisture—silphium.

**Figure 7:**
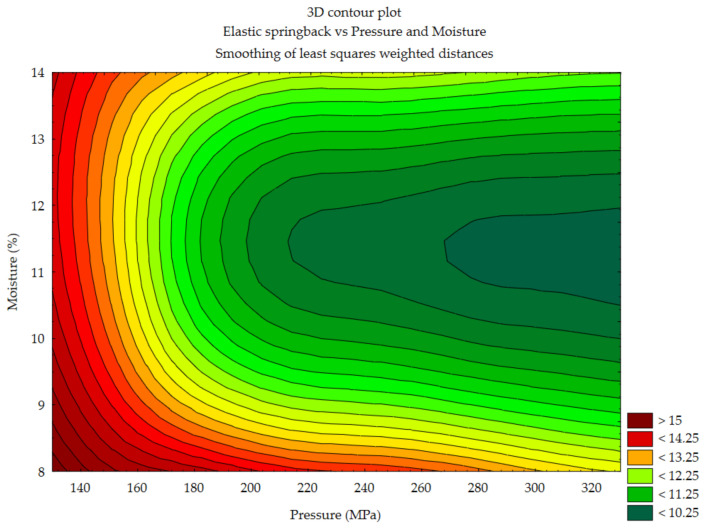
Elastic springback vs. pressure and moisture—sida.

Visualization of the observed changes is presented in the contour plots (Figure 5, Figure 6 and Figure 7) For miscanthus the range of the obtained values of the spread is 9.25–16%. The range of values for the other materials is 10.5–16% (silphium) and 10.25–15% (sida). It can be concluded that the ranges of elastic springback values of the tested materials practically coincide. In the case of miscanthus in the entire range of tested pressure we can observe the minimum values of elastic springback (9–11%) provided that the moisture content of material was at the level of 8%. To obtain the minimum SP at a higher moisture content, it was necessary to increase the compaction pressure at the same time. However, the upper moisture limit for the minimum elastic springback was about 10%.

In case of silphium, obtaining minimal elastic springback range also required material with moisture content of 8% but with pressure above 260 MPa. An increase in pressure resulted in a minimum elastic springback range, but only in material with a moisture content of up to about 9%.

Other changes were observed in the case of sida. Minimum elastic springback values were obtained when the pressure exceeded 190 MPa, but the moisture content of the material had to be in the range of about 10.5–12.5%. Further increase of pressure allowed the range of moisture to extend to 9.5–13%.

The differences and trends noted in the Figure 5, Figure 6 and Figure 7 necessitated additional analyses. Additional contour plots were made showing the changes in elastic springback vs. density and durability and the correlation values of elastic springback vs. density and elastic springback vs. durability.

#### 3.1.5. Elastic Springback vs. Density and Durability

**Figure 8:**
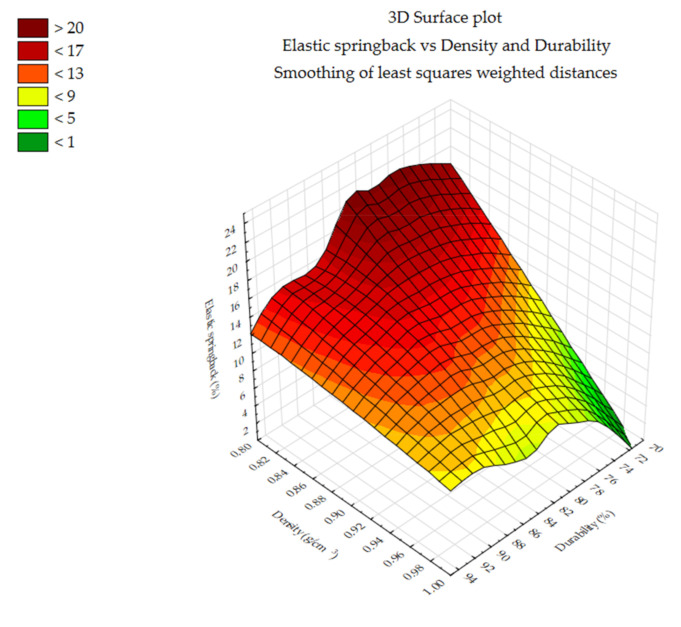
Elastic springback vs. density and durability—miscanthus.

**Figure 9:**
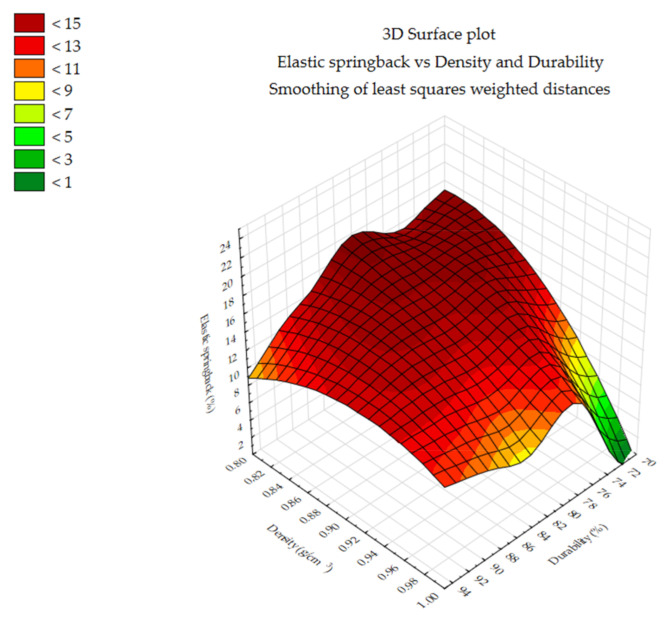
Elastic springback vs. density and durability—silphium.

**Figure 10:**
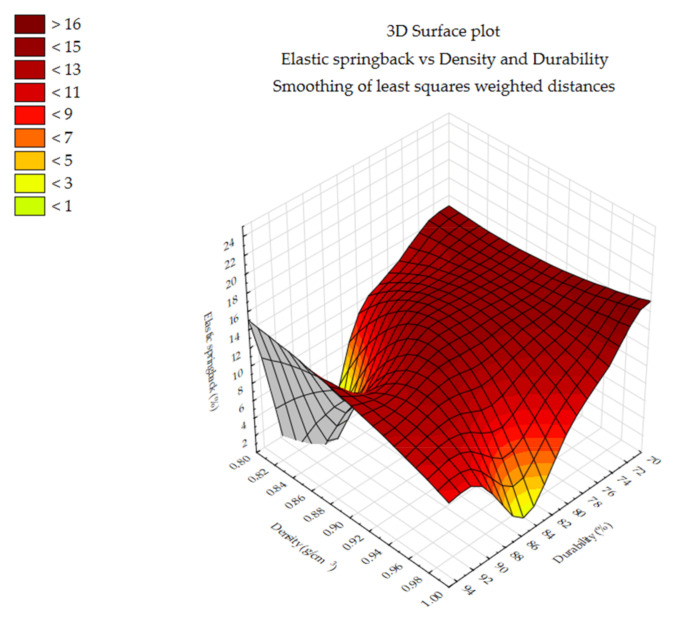
Elastic springback vs. density and durability—sida.

Figure 8, Figure 9 and Figure 10 show the changes in elastic springback vs. density and durability. The values are shown as a contour plot. Elastic springback values were smoothing of least squares weighted distances. Density and durability values are from a study by Styks et al. 2020 [24] performed on the same samples under the same process parameters. For miscanthus and silphium, it can be seen that elastic springback value is related to density.

**Figure 11:**
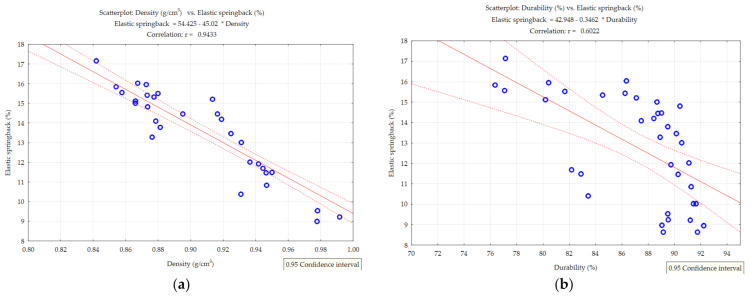

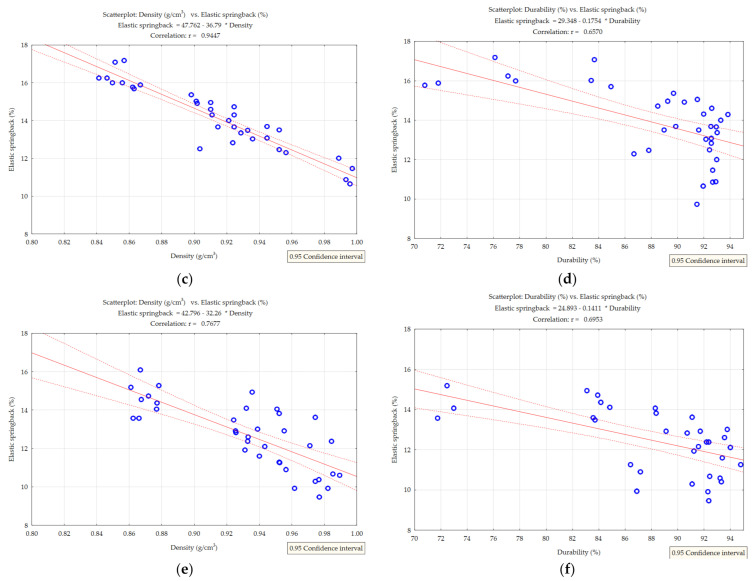
Correlation values. (**a**) Elastic springback vs. density—miscanthus, (**b**) elastic springback vs. durability—miscanthus, (**c**) elastic springback vs. density—silphium, (**d**) elastic springback vs. durability—silphium, (**e**) elastic springback vs. density—sida, and (**f**) elastic springback vs. durability—sida.

Correlation analysis was performed for two variants—density (g/cm^3^) vs. elastic springback and durability vs. elastic springback. Figure 11 plots the correlation values of elastic springback vs. density and elastic springback vs. durability. In the cases of miscanthus and silphium, it can be seen that elastic springback is correlated with density (correlation coefficient miscanthus—r = 0.9433 and silphium—r = 0.9447). This means that the elastic springback has an effect on the density value. Therefore, knowing this, minimizing elastic springback will result in an increase in density without changing the tested parameters (pressure and moisture). Minimization of elastic springback, which requires further research, can be achieved by changing the fineness of the material, adding a binder, or applying a temperature elevation. These changes are aimed at minimizing the elastic stresses in the bed material (proper particle arrangement in the mass, bonding through binder, and plasticization). Knowing this, further study in this direction is necessary.

In the case of sida, the correlation of elastic springback with density was at r = 0.7677, which is lower than the other materials.

The correlation value of elastic springback vs. durability for all plants came out relatively low. This indicates no correlation between elastic springback and durability. However, minimizing elastic springback will not reduce the mechanical durability of the pellet.

The research has made it possible to indicate which process parameters, together with raw material characteristics, produce minimal elastic springback (in the research it was the expansion from 9 to 11%). However, the more important result, and at the same time a new approach to the study of the expansion process, is the indication of where this elastic springback is the largest (reaching up to 16%) because this is where further research should focus to minimize it. These are the ranges of the lowest test pressures and higher moisture contents. Determining the influence of other factors to lower the elastic springback in these areas would allow pellets with high DE and DU to be obtained, but without expenditures on scrubbing to level 8% and creating high pressure.

This is important from a practical point of view as the pressure used in the briquetting process is 50 MPa [55] to 130 MPa [56] in some cases even 250 MPa [57]. Most commonly, the pressure in the pelletizing process is in the range 100–200 MPa but is sometimes more [38]. The zones of high springback values for the tested materials are as follows: for miscanthus the moisture content is above 10% and the pressure is up to about 260 MPa, for silphium the pressure is up to 220 MPa in the whole range of moisture content, and for sida the pressure is up to 170 MPa in the whole range of moisture content.

**Figure 12:**
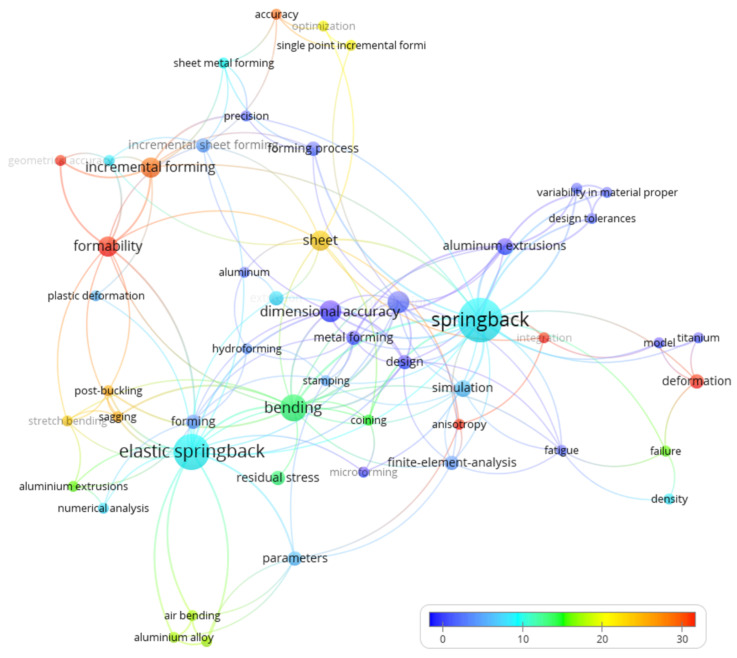
Map of term words with reference to citations.

On the basis of keyword analysis of publications indexed in the Web of Science—Core Collection (Figure 12), the greatest interest of scientists has been shown in geometric accuracy’, ‘incremental forming’, ‘post buckling’, ‘deformation’, and ‘anisotropy’. It can be noted that the research was mainly concerned with changes in the geometry of the agglomerate after a selected time, the influence of various factors on elastic springback. The researchers also discussed computer simulations, modelling, and optimization of compaction process and its influence on elastic springback.

The topic of elastic springback in the area of production of solid biofuels (e.g., pellets) is relatively new, but increasingly important. In the publication Frodeson et al. [47] elastic springback of pellets from cellulose, hemicelluloses, pectin, and two woods at different moisture contents was analyzed. Dhamodaran and Afzal [29] modelled the length of pellets, depending on other parameters (moisture, temperature, pressure, and hold time) and elastic springback of pellets from selected maple hardwood and white spruce softwood species was analyzed. In turn, Wu et al. [58] tested the vibration-assisted compaction of biomass as a method to reduce the energy requirement.

Different values of parameters determining the zones of high springback indicate that its value is significantly affected by material parameters. In the case of miscanthus, the lowest values of pressure allow low springback to be obtained, provided that the moisture content does not exceed 8%. In the case of silphium, the required moisture content is also 8%, but the pressure should be increased to a value above 260 MPa. Sida also requires higher pressures, above 190 MPa, but the range of moisture content is different than in the previous cases and is between 10.5 and 12.5%. These differences are most likely determined by the different chemical composition. As shown in Table 4 of all the materials tested, sida contains the most lignin, almost 20%. As already mentioned, higher temperatures are needed to activate the binding properties of lignin. In our case, most likely the bonding mechanisms were not activated, however, high lignin content could cause the permanent deformation of particles under pressure (reduction of their volume and internal porosity). The influence of material properties on elastic springback was shown by Dhamodaran and Afzal [29], Hard wood has a lower springback compared to soft wood even though the chemical composition of these materials is similar (Table 4). Frodeson et al. [47] explain these differences by different types of hemicelluloses—xylan and mannan. Material with higher xylan content has lower springback because xylan is more affected by water than mannan. However, this requires further research.

Out of the promising factors reducing elastic springback, temperature is most often mentioned, but at the right level and with the right moisture content for the material. The interaction of moisture and temperature causes a lignin–glass transition [28] and as a result particles of the material are joined together. However, taking into account the low lignin content of the investigated materials in comparison to wood this effect may be weaker (Table 4). Another factor may be degree of fragmentation, which lowers the springback value [29]. But unfortunately, both an increase in process temperature and a higher degree of fragmentation require additional expenditures for either grinding and heating.

Another factor can be the drying temperature of the raw material. This process is always one of the steps in the production of solid biofuels and others [63,64], and by applying the right temperature the properties of the raw material can be changed. In the case of torrefaction, this effect is undeniable and improves the conversion by increasing the brittleness of the material [46,65]. A lower temperature range (60–140 °C) [22,66] also affects the grindability of the raw material, which makes it possible to obtain material of a finer grain size with the same, or even lower, input. Moreover, higher brittleness of such material will also be a factor influencing reduction of SP. Thus, there is a group of additional factors which in different ways may interact with the main factors (pressure and moisture content). The influence of these factors and their interactions should therefore be the subject of further research.

## 4. Conclusions

1. These studies have shown the influence of compaction pressure and moisture on post-agglomeration elastic springback. Depending on the features of the material, the influence of compaction pressure and moisture is different. The analysis showed clear differences for all tested raw materials. This may be related to the biological structure of the plant itself and its chemical composition.
2. Analysis of correlation values showed that elastic springback vs. durability correlation is absent for all the plants studied. For the correlation analysis of elastic springback vs. density, the correlation value depends on specific plants. The results showed that elastic springback clearly affects the density (for miscanthus and silphium). This information is very important to optimize the pellet production process.
3. Areas of springback high value in the pattern of process parameters (P, MC) were determined, and these areas indicate directions for further research to minimize springback at these process parameters. In these zones, the influence of other factors such as temperature and degree of defragmentation should be studied.
4. The appearance of elastic springback causes decrease of density. Therefore, material preparation processes and compaction process parameters must strive to minimize the elastic springback. This will result in an improvement of density and certainly not decrease of durability (there was no correlation between elastic springback and durability).

The results and trends indicated should be further developed to determine which processes will minimize this adverse phenomenon occurring during pressure compaction of biomass for energy purposes.

## Figures and Tables

**Table 1:** Two-way analysis of variance (ANOVA)—miscanthus.

	SS	df	MS	F Value	*p*-Value
Intercept	5905.110	1	5905.110	15,096.26	0.000000
Pressure	39.057	3	13.019	33.28	0.000000
Moisture	182.245	2	91.123	232.95	0.000000
Pressure × Moisture	8.821	6	1.470	3.76	0.008861
Error	9.388	24	0.391		

**Table 2:** Two-way analysis of variance (ANOVA)—silphium.

	SS	df	MS	F Value	*p*-Value
Intercept	6998.309	1	6998.309	24,931.12	0.000000
Pressure	77.152	3	25.717	91.62	0.000000
Moisture	31.750	2	15.875	56.55	0.000000
Pressure × Moisture	8.794	6	1.466	5.22	0.001455
Error	6.737	24	0.281		

**Table 3:** Two-way analysis of variance (ANOVA)—sida.

	SS	df	MS	F Value	*p*-Value
Intercept	5809.750	1	5809.750	12,955.44	0.000000
Pressure	47.081	3	15.694	35.00	0.000000
Moisture	40.533	2	20.266	45.19	0.000000
Pressure × Moisture	7.102	6	1.184	2.64	0.041374
Error	10.763	24	0.448		

**Table 4:** Chemical composition of biomass.

Species	Cellulose	Hemicellulose	Lignin	Author
*Miscanthus × giganteus*	41.9 49	16.6 30	13.3 11	[59] [60]
*Silphium perfoliatum*	36	18	12	[60]
*Sida hermaphrodita*	38.3 32.8	46.11 29.3	19.92 19.7	[61] [62]
Hard wood	43.3	31.8	24.5	[59]
Soft Wood	40.4	31.1	28.0	[59]

## Data Availability

Data is contained within the article.
